# Relationships of Educational Attainment and Household Food Insecurity with Obesity: Findings from the 2007–2016 National Health and Nutrition Examination Survey

**DOI:** 10.3390/ijerph18157820

**Published:** 2021-07-23

**Authors:** M. Monique McMillian, Roland J. Thorpe

**Affiliations:** 1 Teacher Education and Professional Development, School of Education and Urban Studies, Morgan State University, Baltimore, MD 21251, USA; 2 Program for Research on Men’s Health, Hopkins Center for Health Disparities Solutions, Johns Hopkins Bloomberg School of Public Health, Johns Hopkins University, Baltimore, MD 21205, USA; rthorpe@jhu.edu

**Keywords:** black young adults, educational attainment, household food insecurity, obesity

## Abstract

This study aimed to determine whether 1882 Black young adults’ educational attainment was associated with their obesity ([BMI] ≥ 30) and whether this association varied with household food insecurity. Data from interviews with Black young adults and a medical examination from the 2007–2016 National Health and Nutrition Examination Survey were analyzed. Modified Poisson regressions with robust standard errors were used. Educational attainment was not associated with obesity (prevalence ratio [PR] = 1.05, 95% confidence interval [CI]: 0.85, 1.30) after adjusting for age, sex, marital status, smoking status, drinking status, income, health insurance status, physical activity level, and household food insecurity. The interaction between educational attainment and household food insecurity was also not significant (PR = 1.11, 95% CI: 0.56, 2.19) after adjusting for the same covariates. These findings indicated that college graduates were as likely to be obese as those with less education, and the relationship between educational attainment and obesity did not vary with household food insecurity. Future studies should conduct longitudinal analyses of these relationships. There is a need to identify the roles that education, household food insecurity, and other measures of socioeconomic status play in Black young adults’ obesity.

## 1. Introduction

Obesity (body mass index [BMI] ≥ 30) among Black adults is a significant public health concern associated with numerous morbidity- and mortality-related diseases, including cardiovascular disease, diabetes, and some cancers [1,2,3]. Non-Hispanic Black adults’ rate of obesity is higher than those of most other racial and Hispanic-origin groups [4]. Among all young adults between the ages of 20 and 39, 60.3% are obese, compared to 71.9% of non-Hispanic Blacks of the same age [4]. Common explanations of obesity include physical inactivity, insufficient nutrition, and social determinants of health, such as socioeconomic status. Educational attainment may partially explain non-Hispanic Black adults’ obesity.

Although educational attainment tends to be inversely related with obesity, studies of how Black adults’ educational attainment relates to their obesity have been inconsistent [5,6]. Some research indicates that Black college graduates are less likely to be obese than those with less education [7]. Some studies indicate that Black college graduates are as likely to be obese as non-college graduates [7,8,9]. Other studies have found no such relationship among Black men, but an inverse relationship among Black women [10,11]. These inconsistent findings suggest the need to examine whether the interaction between educational attainment and other factors is associated with obesity [5].

Research suggests that the interaction between educational attainment and household food insecurity (i.e., insufficient and uncertain availability of nutritious and safe foods) is associated with obesity [12,13]. Some of this research has examined whether the interaction between educational attainment and food availability (e.g., healthy foods in supermarkets and unhealthy foods in fast-food outlets) is associated with obesity [14,15]. Food availability predicts household food insecurity, and the interaction between educational attainment and food availability is associated with obesity [14,15,16]. Unhealthy fast-food outlets are positively associated with obesity in the least-educated adults, but not associated with obesity in the most-educated adults [14]. Similarly, supermarket distance, which tends to decrease access to healthy foods, has a stronger negative association among the least-educated than the most-educated adults [15,17]. Furthermore, the availability of unhealthy foods is associated with increased unhealthy food consumption among the least-educated adults and increases the likelihood of obesity more in the least-educated than in the most-educated adults [14,18]. Given that food availability predicts household food insecurity, the interaction between household food insecurity and educational attainment might predict obesity as well [16].

Pan and colleagues found that in a sample of U.S. adults ranging from young adulthood to old age, the interaction between educational attainment and household food insecurity was associated with obesity [12]. Paradoxically, although household food insecurity does not increase the likelihood of obesity for college graduates, it increases the likelihood of obesity among those with a high school/GED or less [12]. Jointly, educational attainment, food availability, and household food insecurity studies indicate that it is important to determine how educational attainment, household food insecurity, and obesity interrelate.

The objective of the current study was to examine whether Black young adults’ educational attainment and the interaction between their educational attainment and household food insecurity were associated with obesity. Contrary to previous research, this study examined whether household food insecurity moderated the relationship between educational attainment and obesity, rather than whether educational attainment moderated the relationship between household food insecurity and obesity. We hypothesized that college graduates would be less likely to be obese than non-college graduates. Moreover, we hypothesized that there would be an interaction between educational attainment and household food insecurity such that obesity would be higher among Black young college graduates who are food insecure than among Black young college graduates who are food secure.

## 2. Materials and Methods

### 2.1. Sample

The data for this study came from the National Health and Nutrition Examination Survey (NHANES), an ongoing program of nationally representative studies examining children’s and adults’ health and nutrition [19,20,21]. For each two-year cycle, approximately 12,000 civilian, non-institutionalized individuals who had been asked to participate were selected through a stratified, multistage sampling design that included an oversampling of non-Hispanic Blacks, Hispanics, low-income persons, and persons aged 80 and over, among other groups [19,20,21]. Once selected, participants were asked to complete three successive phases: a household interview, a medical examination, and a blood sample. Additional details can be found elsewhere [19,20,21]. The dataset used for the current study included participants from the 2007–2008, 2009–2010, 2011–2012, 2013–2014, and 2015–2016 NHANES studies. Initially, this dataset included a total of 1976 Black young adults, of which 94 were excluded because they were either pregnant or missing BMI or educational attainment data. Our final analytic sample included 1882 Black young adults. 

### 2.2. Study Measures

Obesity: Obesity was the outcome variable used in this study. BMI was calculated based on the height and weight that was measured during the medical examination [2]. Consistently with prior work [22], individuals who had BMI ≥ 30 kg/m^2^ were considered obese. A binary variable was created to identify obese Black young adults (1 = obese; 0 = not obese) [2].

Educational attainment: Educational attainment was the main independent variable. During the home interview, young adults reported their level of education as either less than 9th grade, 9th–11th grade (including 12th grade without a diploma), high school or GED equivalent, some college or an associate’s degree, or a bachelor’s degree or above. These responses were used to create a categorical variable with four levels: college graduate, some college, high school/GED, and less than high school [12]. 

Household food insecurity: Household food insecurity, the moderator examined in this study, was based on the 10-item United States Department of Agriculture Food Security Survey Module, which measured the overall household food insecurity status and did not include child-specific questions [23]. Respondents’ affirmative answers (i.e., “yes”, “often”, “sometimes”, “almost every month”, and “some months but not every month”) to the following questions were coded as 1: (1) whether household members were worried they would run out of food, (2) whether food did not last, (3) whether the household could not afford balanced meals, (4) whether adult household members cut the size of or skipped meals, (5) how often adults cut or skipped meals, (6) whether members of the household ate less than they should, (7) whether members of the household were hungry but did not eat, (8) whether they lost weight because there was no money for food, (9) whether adults in the household had not eaten for a whole day within the last 12 months, and (10) approximately how often adults had not eaten for a day. Each affirmative answer was summed for a total score that was then converted into a binary variable to indicate household food insecurity (1 = total score was between 3 and 10) or household food security (0 = total score was between 0 and 2).

Covariates: The covariates used in this study were based on research indicating that educational attainment and household food insecurity were associated with obesity [5,24]. All covariates were self-reported during the home interview and included age (in years), sex (1 = male; 0 = female), marital status (1 = married; 0 = not married), smoking status (1 = current smoker; 0 = not a current smoker), drinking status (1 = current drinker; 0 = not a current drinker), income (below $35,000 as the reference category, in addition to three dummy variables for $35,000 to $74,999, over $75,000, and missing data), insurance status (1 = insured; 0 = not insured), and physical activity level. Consistently with prior work [25], physical activity level was determined based on the individual experiencing at least two periods of activity in the past 30 days; at least one period had to be of moderate intensity that slightly increased breathing or heart rate for at least 10 min continuously (e.g., brisk walking), and at least one period had to be of high intensity that caused a large increase in breathing or heart rate for at least 10 min continuously (e.g., running) [26,27]. Those who completed both periods of activity were rated 1; those who did not were rated 0 [24].

### 2.3. Statistical Analysis

Means and the corresponding standard errors were calculated for continuous variables, and proportions were calculated for categorical variables. The mean and proportional differences among education subgroups were calculated using Student’s t-tests for continuous variables and chi-square tests for categorical variables. A modified Poisson regression with robust standard errors was used in two models because the outcome (i.e., obesity) was greater than 10% [22,28,29]. Model 1 examined the association between educational attainment and obesity after adjusting for household food insecurity and the aforementioned covariates. Model 2 examined whether the relationship between educational attainment and obesity varied with household food insecurity after adjusting for the aforementioned covariates. Taylor linearization procedures and weights were used to account for the complex, multistage sampling design [19,20,21]. *p* values ≤ 0.050 were considered statistically significant. All analyses were conducted using STATA 16.0 [30]. 

## 3. Results

Table 1 summarizes the distributions of demographics, household food insecurity, and obesity in the total sample and according to educational attainment. Among the 1882 young adults, 16.7% were college graduates, 39.4% had completed some college, 27.1% had a high school/GED, and 16.6% had completed less than high school. Approximately 27.6% were household food insecure. The average age was 29.0 (SE = 0.1) years. Approximately 47.6% were male, and 36.0% were married. Around 26.2% were current smokers, and about 71.5% were current drinkers. Approximately 45.8% of the sample had an income of $35,000 or less. Approximately 66.2% were insured; about 56.4% were at least moderately physically active. Around 43.7% of the sample were obese. There were no differences across education subgroups in either obesity (*p* = 0.143) or drinking status (*p* = 0.589). However, there were differences across education subgroups in the remaining variables (*p* ≤ 0.050).

Table 2 indicated whether there was a negative association between educational attainment and obesity and whether this association varied with household food insecurity. Model 1 indicated that young adults who were college graduates were not significantly less likely to be obese than those with less education (prevalence ratio [PR] = 1.05, 95% confidence interval [CI]: 0.85, 1.30). Model 2 indicated that the interactions between each education subgroup and household food insecurity were not significant after adjusting for the same covariates (PR = 1.11, 95% CI: 0.56, 2.19).

## 4. Discussion

This study sought to examine the association between educational attainment and obesity and the effect of the interaction between educational attainment and household food insecurity on obesity among Black young adults between the ages of 20 and 39. Using data from NHANES, after adjustments, we found neither a relationship between educational attainment and obesity nor an association of the interaction between educational attainment and household food insecurity with obesity. College graduates were as likely to be obese as non-college graduates, and the interplay between educational attainment and household food insecurity did not change this likelihood. These findings suggest that Black young adults’ educational attainment and the interaction between educational attainment and household food insecurity are not related to obesity. Given that obesity is higher among non-Hispanic Black adults than most other racial groups, understanding how educational attainment, household food insecurity, and obesity interrelate could lead to strategies for preventing it.

Although our finding that educational attainment was not negatively associated with obesity was unexpected, it is consistent with some research [8]. Some studies have found that Black young adults who are college graduates are as likely to be obese as non-college graduates [5,8]. A possible reason for our finding is that our study included sex as a covariate rather than running the analyses by sex. Studies that have focused on one sex or that were stratified by sex found no relationships between educational attainment and obesity among men, but an association among women [9,10]. Furthermore, the current study measured educational attainment in terms of years of education—namely, college graduates, some college, high school/GED, and less than high school. However, there might be differences in sex that are related to other qualities of educational attainment, such as college major, which would warrant analysis by sex [31]. For example, evidence suggests that the industrial arts/recreation major is associated with poor health in men, but not women [31]. Thus, the relationship between educational attainment and obesity might be significant if the college major were considered as a measure of educational attainment. Additional analyses are needed to determine whether the relationship between Black young adults’ educational attainment and obesity is significant if analyses are conducted by sex and educational attainment is measured by major. 

The finding that the interaction between educational attainment and household food insecurity is not associated with obesity is inconsistent with a previous study that examined this relationship in U.S. adults with a broader age span and from various ethnic backgrounds [12]. A likely explanation is that our study focused exclusively on Black young adults between the ages of 20 and 39—a time that may have more life-course transitions than any other period [32]. We considered one of these transitions by excluding pregnant women in the current analysis; however, other life transitions related to obesity might have contributed to our findings [32]. It is possible that acute household food insecurity during young adulthood interacts differently with educational attainment than chronic household food insecurity. However, current measures of household food insecurity do not do a good job of differentiating between chronic and acute household food insecurity [33]. Perhaps the interaction between chronic household food insecurity when a young adult is living alone and has just graduated from college is different from acute household food insecurity when that adult has married and has a newborn. 

This study has several strengths and limitations. Two of the strengths are that the data were nationally representative and included multiple years. Thus, the findings related to educational attainment and the interaction between educational attainment and household food insecurity are generalizable to Black young adults between the ages of 20 and 39. In addition, using multiple years of data increased the statistical accuracy of our estimates. However, the current study was cross-sectional; therefore, we could not examine causality. In addition, we used a dichotomous measure of household food insecurity. Because our household food insecurity measure was dichotomous, we could not distinguish between high versus moderate household food insecurity or between low versus very low household food insecurity.

## 5. Conclusions

In conclusion, we found that neither educational attainment nor the interaction between educational attainment and household food insecurity was associated with obesity. These findings indicate that educational attainment is not independently related to obesity among non-Hispanic Black young adults after taking into account demographic factors and health behaviors; furthermore, this relationship is not modified by household food insecurity. More research is needed to determine whether the relationship between Black young adults’ educational attainment and obesity is modified by other social determinants of health.

## Figures and Tables

**Table 1:** Demographics, educational attainment, household food insecurity, and obesity among Black young adults of ages 20–39; NHANES 2007–2016.

Variable	Total Sample (*N* = 1882)	Less Than High School (*N* = 313)	High School/GED (*N* = 511)	Some College (*N* = 743)	College Graduate (*N* = 315)	*p* Value
Age (years) mean ± SE	29.0 ± 0.1	29.0 ± 0.4	28.6 ± 0.4	28.4 ± 0.4	31.0 ± 0.3	<0.001
Male, *n* (%)	932 (47.6)	170 (53.4)	287 (53.9)	342 (43.9)	133 (40.4)	<0.001
Married, *n* (%)	685 (36.0)	121 (37.9)	170 (32.7)	258 (34.2)	136 (43.8)	<0.050
Current Smoker, *n* (%)	503 (26.2)	150 (48.3)	164 (31.1)	164 (22.0)	25 (07.5)	<0.001
Current Drinker, *n* (%)	1174 (71.5)	198 (72.7)	310 (68.9)	468 (73.0)	198 (71.0)	0.589
Educational Attainment, *n* (%)						
Less Than High School	313 (16.1)					
High School/GED	511 (26.9)					
Some College	743 (39.7)					
College Graduate	315 (17.1)					
Income, *n* (%)						<0.001
<$35,000	863 (45.8)	209 (66.6)	277 (54.4)	310 (42.2)	67 (21.2)	
$35,000–$74,999	639 (33.8)	75 (24.1)	168 (32.7)	275 (36.2)	121 (38.9)	
≥$75,000	324 (17.2)	23 (07.2)	51 (09.9)	134 (18.1)	116 (35.8)	
Missing	56 (03.0)	6 (01.9)	15 (02.8)	24 (03.3)	11 (03.9)	
Insured, *n* (%)	1247 (66.2)	183 (57.8)	288 (55.7)	514 (69.4)	262 (83.2)	<0.001
Physically Activity, *n* (%)	1065 (56.4)	131 (41.8)	259 (49.6)	436 (58.8)	239 (75.5)	<0.001
Obese, *n* (%)	813 (43.7)	125 (39.7)	215 (41.3)	334 (46.4)	139 (44.7)	0.143
Household Food Insecure, *n* (%)	523 (27.6)	119 (38.3)	154 (30.5)	205 (27.3)	45 (14.1)	<0.001

Note. Chi-square tests were run to determine the proportions among the education subgroups. Student’s t-tests were run to determine the mean differences among subgroups.

**Table 2:** Associations of educational attainment and household food insecurity with obesity among 1607 Black young adults (ages 20–39); NHANES 2007–2016.

	Model 1	Model 2
Variable	Prevalence Ratio (95% CI)	Prevalence Ratio (95% CI)
Age	1.03 (1.02, 1.04)	1.03 (1.02, 1.04)
Male	0.63 (0.56, 0.72)	0.63 (0.56, 0.72)
Married	1.15 (1.02, 1.29)	1.15 (1.02, 1.29)
Current Smoker	0.92 (0.80, 1.06)	0.92 (0.80, 1.06)
Current Drinker	1.08 (0.93, 1.24)	1.08 (0.93, 1.24)
Educational Attainment		
Less Than High School	1.05 (0.85, 1.30)	1.03(0.80, 1.35)
High School/GED	1.09 (0.92, 1.30)	1.09(0.88, 1.34)
Some College	1.15 (1.00, 1.33)	1.13 (0.96, 1.33)
College Graduate	Ref.	Ref.
Income		
<$34,999	Ref.	Ref.
$35,000–$74,999	0.90 (0.80, 1.02)	0.90 (0.80, 1.02)
Over $75,000	0.94 (0.81, 1.08)	0.94 (0.81, 1.08)
No data	1.00 (0.69, 1.45)	1.00 (0.69, 1.45)
Insured	1.11 (0.96, 1.28)	1.11 (0.96, 1.28)
Physically Active	1.03 (0.90, 1.17)	1.03 (0.90, 1.17)
Household Food Insecurity	1.00 (0.87, 1.14)	0.91 (0.87, 1.14)
Household Food Insecurity * Less Than High School		1.11 (0.56, 2.19)
Household Food Insecurity * High School/GED		1.07 (0.58, 1.96)
Household Food Insecurity * Some College		1.13 (0.61, 2.08)
Household Food Insecurity * College Graduate		Ref.

Note. Model 1 adjusted for age, sex, marital status, smoking status, drinking status, income, household food insecurity, and physical activity. Model 2 included education subgroups with household food insecurity two-way interaction terms. CI = confidence interval. The “*” indicates the interaction between the two variables.
